# Tumor-matched and unmatched cancer associated fibroblasts exhibit differential effect on proliferation and FMOD and MMP9 gene expression in head and neck squamous cell carcinoma cells when cocultured in spheroids

**DOI:** 10.1186/s12935-024-03388-0

**Published:** 2024-05-31

**Authors:** Max Rademaekers, Emil Oliver Johansson, Ellen Johansson, Karin Roberg, Emilia Wiechec

**Affiliations:** 1 Division of Cell Biology, Department of Biomedical and Clinical Sciences, Linköping University, Linköping, Sweden; 2 Department of Otorhinolaryngology, Region Östergötland, Linköping, Sweden

**Keywords:** Cancer-associated fibroblasts, Head and neck squamous cell carcinoma, 3D cultures, Proliferation, Cisplatin, Drug response

## Abstract

**Background:**

Cancer-associated fibroblasts (CAFs) are the major cellular component of the tumor microenvironment and are known to affect tumor growth and response to various treatments. This study was undertaken to investigate the crosstalk between tumor-matched or unmatched CAFs and head and neck squamous cell carcinoma (HNSCC) cells regarding tumor growth and treatment response.

**Methods:**

Three HNSCC cell lines (LK0412, LK0902 and LK0923), were cocultured in 2D or in 3D with their tumor-matched CAFs, site matched CAFs from other tumors or normal oral fibroblasts (NOFs). Cell proliferation was assessed as the amount of Ki67 positive cells/ spheroid area in formalin-fixed- paraffin-embedded 3D spheroids stained with Ki67 antibody. Viability after seven days of cisplatin treatment was measured with CellTiter-Glo 3D Viability Assay. The mRNA expression of CAF-associated markers (*ACTA2, COL1A2, FAP, PDGFRα, PDGFRβ, PDPN, POSTN* and *S100A4*) in CAFs before and after coculture with tumor cells as well as mRNA expression of CAF-induced genes (*MMP1*, *MMP9* and *FMOD*) in tumor cells separated from CAFs after co-culture was measured with RT-qPCR. The expression of selected protein biomarkers was validated with immunohistochemistry based on previous mRNA expression results.

**Results:**

The proliferation of the LK0412 and LK0902 tumor spheroids varied significantly when cocultured with different CAFs and NOFs as shown by Ki-67 positive cells. RT‒qPCR analysis revealed different molecular profile of the analyzed HNSCC-derived CAFs concerning the expression of CAF-associated markers. The interaction between CAFs and HNSCC cells was more pronounced after coculture with unmatched CAFs as shown by changes in mRNA expression pattern of CAF-specific markers. Additionally, the unmatched CAFs significantly upregulated the mRNA expression of *MMP1*, *MMP9* and *FMOD* in tumor cells compared to tumor-matched CAFs.

**Conclusion:**

Our results indicate that tumor-matched CAFs are unique for each tumor and affect the proliferation and the gene/protein expression of tumor cells in a distinct manner. The interaction between tumor unmatched CAFs and HNSCC cells in the tumor spheroids is associated with significant changes in the mRNA expression of CAF-specific markers and significant increases in FMOD and MMP9 in tumor cells compared to when cocultured with tumor-matched CAFs. Taken together, our results show how important the selection of CAFs is to get a reliable in vitro model that mimics the patients’ tumor.

## Background

Head and neck squamous cell carcinoma (HNSCC) is the seventh most common cancer form worldwide, accounting for approximately 5% of all cancer cases, and originates from the mucous membranes of the oral cavity, pharynx, larynx and nasal cavity [1]. The five-year overall survival for all HNSCC sites combined is approximately 60%, with great variance between the different subgroups of the disease, disease stage at time of diagnosis and socioeconomic standard [2].

In the struggle to understand malignant tumors, it is crucial to understand not only malignant cells but also the tumor microenvironment (TME), which comprises many different cell types in complex interactions with each other and the extracellular matrix (ECM) [3]. Among these are cancer-associated fibroblasts (CAFs), which have been proven in several studies to affect tumor growth, epithelial-to-mesenchymal transformation (EMT), metastatic potential and local invasiveness, not only in HNSCC but also in various groups of cancer [4, 5]. A dense tumor stroma, i.e. a high CAF fraction, is linked to an increased capacity for vascular and perineural invasion, which in turn is associated with recurrent disease and a lower overall survival [6]. Recent findings revealed that upregulation of the CAF-regulated genes *MMP9* and *FMOD*, which are important for the remodeling of the ECM, has been associated with improved overall survival (OS) in patients treated with radiotherapy [7]. However, it is also believed that a high production of proteins regulating ECM stiffness predisposes patients to local invasiveness, metastatic potential, and risk of recurrence [8], highlighting the complexity of the tumor microenvironment.

CAFs constitute an attractive therapeutic target, but the heterogeneity and complexity of CAFs pose challenges. Recent studies have revealed the extent of CAF heterogeneity and the role of various subtypes in cancer progression [9]. The progression of HNSCC has been linked to distinct subtypes of CAFs that present functional heterogeneity and have differential tumor-promoting capabilities [10, 11].

Moreover, the protumorigenic function of CAFs is expressed by their capability to induce chemo- and radioresistance in vitro and in vivo; however, the mechanism remains elusive [12, 13]. In contrast to the protumorigenic effect of CAFs, there are few reports supporting their antitumorigenic properties. Therefore, further studies on the usefulness of CAF targeting for therapeutic purposes are indispensable [14–17].

The culturing of cells in 3D tumor spheroids has been shown to replicate the in vivo circumstances more accurately in an in vitro setting compared to standard 2D culture. This tumor model is steadily gaining recognition in the scientific community, not only in the field of HNSCC [18, 19]. A multitude of studies indicate that a more lifelike pattern of cell‒cell interaction, tumor morphology, signal transduction and differentiation contributes to this and subsequently makes the results from studies using this method more likely to be indicative of in vivo replicability [19, 20]. In a previous study using a unique 3D model consisting of head and neck tumor cells and tumor-matched CAFs, we showed that CAFs impact proliferation, cisplatin and cetuximab sensitivity and the EMT and cancer stem cell phenotype of tumor cells [18]. Furthermore, CAF-derived signals induced the expression of multiple genes involved in proliferation, differentiation, and metastasis in the 3D tumor model. Additionally, high mRNA expression of the two CAF-induced genes, namely, *MMP9* and *FMOD*, was found to be associated with better overall survival in HNSCC patients treated with radiotherapy [7].

The aim of the present study was to further investigate the effect of the direct interaction between NOFs, tumor-matched or unmatched CAFs with tongue and laryngeal tumor cells in terms of phenotype, cell proliferation and treatment response when cocultured in 3D.

## Materials and methods

### Tumor samples and cell lines

Samples from patients with HNSCC were obtained from an established tumor collection (No 416, The National Board of Health and Welfare in Sweden) at the Department of Otorhinolaryngology, Head and Neck Surgery, at the University Hospital of Linköping, Sweden. The Ethical Committee of Linköping approved the collection (approval no. 03-537), and written consent was acquired from the patients. Tumor cell lines and CAFs were established from pretreatment HNSCC biopsies as previously described [4, 21]. All CAF cultures showed homogenous, positive immunofluorescent staining for vimentin and negative for cytokeratin [4]. Normal oral fibroblasts (NOFs) were established from biopsies harvested during benign surgery, mainly tonsillectomies, at the Department of Otorhinolaryngology, Head and Neck Surgery, at the University Hospital of Linköping, Sweden (approved by the Linköping University ethical committee). The genetic compatibility of the above described HNSCC cell lines with the tumor tissue from which a given cell line originates has been confirmed using short tandem repeat (STR) analysis with the GenePrint® 10 System (Promega, USA).

### Cell culture

HNSCC cell lines (LK0902, LK0923 and LK0412), CAF cultures (0902, 0923, 0820, 0836, 0843, 0861, 1001, 1002) (Table 1) and NOFs from four different patients (NOF1, NOF2, NOF3 and NOF4) were used in this study. The HNSCC cell lines and CAFs were cultured in keratinocyte-SFM supplemented with antibiotics (penicillin 50 U/ml, streptomycin 50 U/ml) and 10% FBS (all from GIBCO, Invitrogen Corporation, Paisly, UK). The media were changed twice a week, and the cells were subcultured weekly after detachment with 0.25% trypsin + 0.02% EDTA at a split ratio of approximately 1:2 or 1:3. The cultures were routinely checked for mycoplasma contamination using the MycoAlert Mycoplasma Detection Kit (Lonza, Walkersville, MD USA) [21].

**Table 1:** Origin and tumor characteristics of HNSCC cell lines and CAF

Cell line	Gender	Localization	Clinical stage^3^
LK0412	F	Tongue	T1N0M0
LK0902	F	Tongue	T1N0M0
LK0923	F	Larynx	T1N0M0
CAF			
0820CAF	M	Larynx	T4N0M0
0836CAF	M	Larynx	T2N0M0
0843CAF	M	Tongue	T2N0M0
0861CAF	M	Tongue	T1N0M0
0902CAF	F	Tongue	T1N0M0
0923CAF	F	Larynx	T1N0M0
1001CAF	M	Larynx	T1N0M0
1002CAF	F	Tongue	T2N1M0

^a^TNM classification according to the International Union against Cancer (UICC, 2002), female (F), male (M)

### Establishment of 3D cocultures

CAFs and NOFs in passages 4–8 and HNSCC cells in passages 10–25 were used for coculturing. Tumor cells (TCs) were mixed with CAFs at a ratio of 2:1 (approximately 10,000 TCs and 5,000 CAFs/NOFs) and subsequently seeded into 96-well ultralow attachment (ULA) round-bottom plates (Corning, Amsterdam, The Netherlands) to form spheroids.

### Cisplatin treatment of spheroids and viability assay

For treatment, spheroids were cultured for 48 h before the addition of cisplatin (1.5–3 µg/ml; Cisplatin Meda, Sandoz A/S, Denmark) to selected spheroids for seven days. Untreated spheroids were used as controls. Thereafter, viability was measured via CellTiter-Glo® 3D Viability Assay. Briefly, 100 µl of the culture medium was removed and 100 µl of the reagent were added to each well in the 96 ULA plate and then shaken with an Eppendorf shaker at 750 rpm at 22 °C for 5 min, and thereafter, spheroids were transferred to a 96-well plate (Nunclon™ Delta Surface, NuncA/C, Roskilde, Denmark). After 25 min, the viability was measured via luminescence using a Victor3™ plate reader (EG&G Wallac, Upplands Väsby, Sweden).

### Immunohistochemistry (IHC)

For IHC, spheroids were cultured for seven days and then harvested and fixed in 4% paraformaldehyde (Santa Cruz Biotechnology, Dallas, TX, USA) overnight at 4 °C. Thereafter, the cells were rinsed with PBS, stained with 0.1% toluidine blue D (Merck, Kenilworth, NJ, USA) and centrifuged in 2% agarose to form agarose blocks with multiple spheroids. The blocks were next dehydrated and embedded in paraffin wax (Merck, Kenilworth, NJ, USA). Sections of 5 μm were collected on Super Frost Plus slides (Thermo Fischer Scientific, Fremont, CA, USA), dried overnight, and incubated at 58 °C for 60 min. The sections were deparaffinized in Histolab-Clear (Histolab, Gothenburg, Sweden) and rehydrated in alcohols. Antigen retrieval (PT 200, DAKO Denmark A/S) was performed, and sections were blocked for endogenous peroxidase activity in 3% H_2_O_2_ (Sigma‒Aldrich, St. Louis, MO, USA) and thereafter incubated in blocking buffer (0.1% BSA-5% FBS in TBS-Triton). Sections were incubated overnight at 4 °C with primary antibodies: a rabbit polyclonal Ki67 antibody (1:50; Santa Cruz Biotechnology), a rabbit monoclonal MMP9 antibody (1:200, Abcam, Cambridge, UK) and a rabbit polyclonal fibromodulin antibody (1:50; Invitrogen, Carlsbad, CA, USA). The sections were washed in TBS-Triton buffer solution and incubated with goat anti-rabbit secondary antibody (1:500; IgG, EMD Millipore Corporation, Temecula, CA, USA) for 60 min at room temperature. After washing, slides were developed using the ImmPACT NovaRED peroxidase substrate kit (Vector laboratories, Burlingame, CA, USA) for 4 min at room temperature. Sections were counterstained in Mayer’s hematoxylin (Histolab) for 3 min, dehydrated, cleared in Histolab-Clear and finally covered with Pertex (Histolab) and a cover slip. For negative controls, the same procedure as above was used but without applying the primary antibody. These were revealed to be unstained. Images were acquired with a light microscope (Olympus BX51, Shinjuku City, Tokyo, Japan) with a 10x or a 20x objective.

### Image acquisition and quantification of Ki67-positive cells

Images were acquired with a light microscope (Olympus BX51, Shinjuku City, Tokyo, Japan) with a 10x objective. The Ki67-positive cells were counted in at least four images from two independent experiments. Spheroids within the analyzed area were marked manually with a polygon selection tool using ImageJ 1.47 v software (National Institutes of Health, Bethesda, MD). Next, the pixel values of the marked spheroids were measured by the software, which accounts for the area in which Ki67 positive cells were counted.

QuPath 0.3.2 v software (QuPath developers, The University of Edinburgh) with the positive cell detection command was used for quantification of Ki67 positive cells. Images were imported, and the spheroids were marked with the “Brush” tool, carefully avoiding marking debris that could be registered as false positives. The “Positive cell detection” function was used, set to a single threshold detection of the mean optical density (OD) value of the nucleus. As a baseline, this detection value was set to 0.13, and the precision of the cell count was verified manually in 10% of the analyzed images. The results are presented as Ki67-positive cells/area.

### Magnetic activated cell sorting (MACS)

Tumor cells and CAFs were cocultured in 75 cm^2^ cell culture flasks (Corning) at a ratio of 1:1 for seven days and thereafter separated via magnetic activated cell sorting (MACS) as previously described [7]. Briefly, cells growing in 2D were incubated with trypsin, flushed in culture media, centrifuged, washed in PBS, passed through a 50 μm pre-separation filter and then labeled with CD326 (EpCAM) MicroBeads (Miltenyi Biotec, Germany) followed by a 30-min incubation at 4 °C. The cells were washed with PBS twice and subjected to a magnetic field. The epithelial cells adhered to magnetic beads were separated from CAFs (unlabeled cell fraction) using MACS® Column Beads (Miltenyi Biotec) separation system. The procedure was repeated in three separate columns for each cell suspension to minimalize the risk of insufficient separation. Thereafter, separated tumor cells and CAFs were used for mRNA analysis.

To check the purity of the tumor cell/CAFs suspensions, cells were cultured for 48 h and thereafter 500 cells with epithelial or fibroblast morphology were counted in a phase contrast light microscope. Less than 1% of the cells were found with a fibroblast/epithelial morphology in all samples.

### RT‒qPCR

RNA was extracted from TCs, CAFs, and NOFs with the RNeasy Mini Kit (Qiagen, Hilden, Germany), and the obtained RNA was then reverse transcribed into cDNA with the High-Capacity RNA-to-cDNA kit (Applied Biosystems, MA, USA) according to the manufacturer’s instructions using the LifeTouch thermal cycler (Bioer Technology, Zhejiang, China). Real-time quantitative reverse transcription PCR (RT‒qPCR) analysis was performed on a QuantStudio™ 7 Flex Real-Time PCR system (Applied Biosystems, Waltham, MA, USA) using TaqMan® Gene Expression assays (*ACTA2*/Hs00426835_m1; *COL1A2*/Hs01028956_m1; *FAP*/Hs00990807_m1; *PDGFRα*/Hs00998018_m1; *PDGFRβ*/Hs01019589_m1; *POSTN*/Hs00170815_m1; *PDPN*/Hs00366766_m1; *S100A4*/Hs00243202_m1; *MMP1*/Hs00899658_m1; *MMP9*/Hs00957555_m1; *FMOD*/Hs00157619_m1) and amplified using a TaqMan real-time PCR protocol according to the manufacturer’s instructions (Thermo Fisher, Waltham, USA). Briefly, the following thermal profile was used for PCR amplification: 95 ºC for 10 min, 40 cycles of 95 ºC for 15 s and 60 ºC for 60 s. To serve as an internal control, the reference gene β-actin (Hs99999903_m1; Applied Biosystems) was amplified and data from the samples were normalized against this reference gene. The data were calculated according to the comparative Ct method to present the data as fold differences in the expression levels relative to NOFs (average of four different NOFs), which served as control sample.

### Statistical analysis

Statistical analysis was performed using Prism 9.0 (GraphPad Software, Inc., La Jolla, CA, USA). All values obtained are presented as the mean ± SD of at least two (IHC) or three (qPCR) independent experiments. Differences between two groups were analyzed with the unpaired Student’s t-test. The multiple comparison analysis was performed using one-way ANOVA followed by Bonferroni adjustment. A p-value < 0.05 was considered as statistically significant (*).

## Results

### The impact of CAFs on tumor cell proliferation

Our recently published data show that tumor-matched CAFs increased the proliferation of HNSCC cells when cocultured in tumor spheroids compared to HNSCC cells grown alone in spheroids [18]. Here, we aimed to investigate whether NOFs and different site matched CAFs from tongue and larynx cancer affect tumor cell growth in tumor spheroids in a similar way as tumor-matched CAFs. The tumor cell lines LK0412, LK0902 and LK0923 were cocultured with tumor-matched CAFs (not LK0412), CAFs from the same site (unmatched; tongue or larynx) and/or NOFs in 3D for seven days. The proliferation rate was measured on sections stained with a Ki67 antibody by calculation of the number of Ki67^+^ cells/area.

**Fig. 1:**
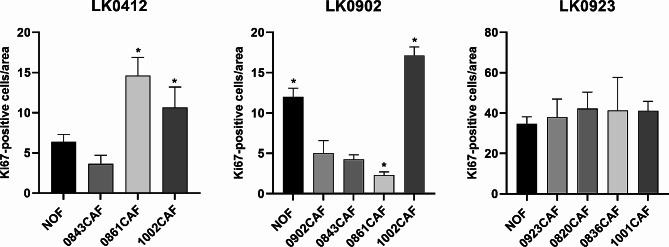
Quantification of Ki67 expression in HNSCC spheroids. Immunohistochemical staining of tumor spheroids with the proliferation marker Ki67 was performed on LK0412, LK0902 and LK0923 spheroids consisting of tumor cells and CAFs from different tumors or NOFs. Quantification of Ki67 positive cells was performed in 7-day-old tumor spheroids; data are depicted as Ki67 positive cells/area ± SD, 2 experiments, images = *n* ≥ 4. *p < 0.05 according to one-way ANOVA with Bonferroni adjustment

In the two tongue cancer cell lines (LK0412 and LK0902), we found that proliferation varied between 3D cocultures with different tongue cancer-derived CAFs and NOFs. A significant increase in cell proliferation was shown in spheroids composed of LK0412/0861CAF and LK0412/1002CAF compared to NOFs (Fig. 1). Unfortunately, the patient-matched 0412CAF was not available for the study. However, we found it interesting to explore whether the same tongue cancer-derived CAFs (0843CAF, 0861CAF and 1002CAF) will have similar effect on another tongue cancer cell line (LK0412). Additionally, we observed a significant decrease in cell proliferation in LK0902 tumor spheroids consisting of tumor-matched CAFs, LK0902/0843CAF and LK0902/0861CAF compared to spheroids with LK0902/NOF. In LK0902/1002CAF cocultures, significantly increased cell proliferation was found (Fig. 1). Interestingly, in LK0902 cells, higher cell proliferation was observed in cocultures consisting of LK0902/NOF than in cocultures consisting of LK0902 tumor-matched CAFs.

In LK0923, no significant differences between various larynx cancer-derived CAFs and NOFs concerning cell proliferation in 3D co-cultures were found. Moreover, co-cultures with LK0923 and larynx cancer-derived CAFs/NOFs exhibited a higher proliferation rate than co-cultures of LK0412 and LK0902 with tongue-derived CAFs (Fig. 1).

### The impact of unmatched CAFs on the tumor response to cisplatin

**Fig. 2:**
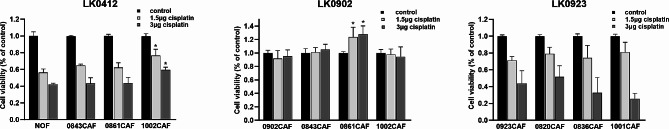
Measurement of cisplatin treatment response. LK0412, LK0902 and LK0923 spheroids composed of tumor cells and CAFs from different tumors or NOFs were treated with cisplatin. After seven days, viability in response to treatment was measured via the CellTiter-Glo 3D Viability Assay. *n* = 12–16 spheroids, *p < 0.05 according to one-way ANOVA with Bonferroni adjustment

We have recently shown that CAFs have an impact on the cisplatin treatment response (measurement of proliferation) when cocultured with tumor cells in spheroids compared to tumor cells alone [18]. Based on our findings and results from previous experiments, we were interested in how tumor-unmatched CAFs modulate the response to treatment in a 3D coculture model compared to matched CAFs or NOFs. The LK0412, LK0902 and LK0923 cell lines were cocultured with different CAFs and NOFs in the same combination as described above, and the response to cisplatin was investigated by means of cell viability. Spheroids with cocultures with LK0412 and LK0923 showed a clear treatment response to both 1.5 and 3 µg/ml cisplatin compared to the unaffected cocultures with LK0902 (Fig. 2). A significantly decreased response to cisplatin was observed in LK0412/1002CAF spheroids compared to cocultures with NOFs (Fig. 2). Similar findings were not seen in the cisplatin-resistant cell line LK0902 cocultured with 1002CAF, which points to a tumor-specific rather than tongue cancer-specific treatment response. Moreover, in LK0902 spheroids, the presence of 0861CAFs was associated with higher cell viability following cisplatin treatment compared to tumor-matched CAFs. No significant changes in the viability of cells treated with cisplatin were observed between cocultures of LK0923 cells with tumor-matched and unmatched CAFs (Fig. 2).

### Expression of CAF-associated markers in HNSCC-derived CAFs

**Fig. 3:**
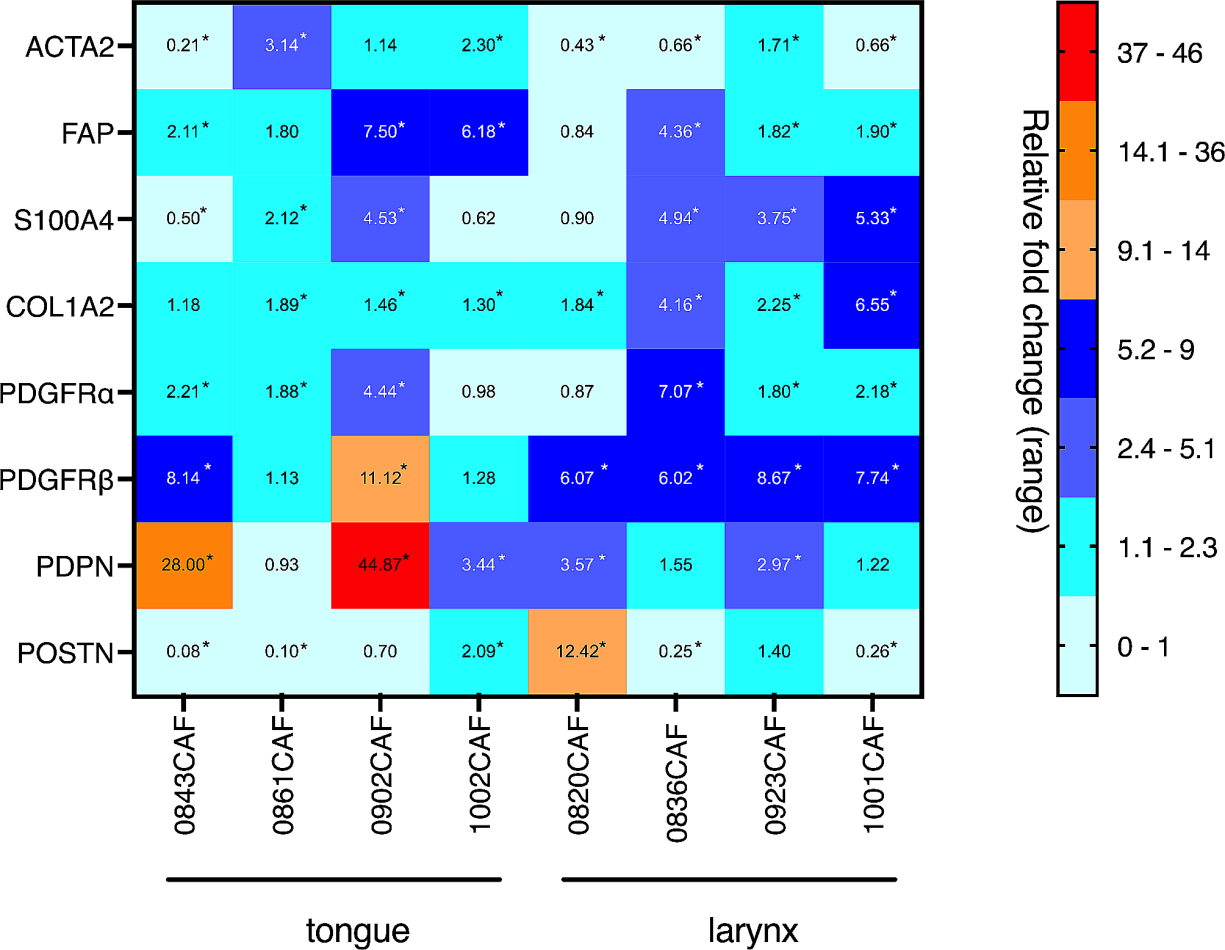
mRNA expression pattern of CAF-specific markers in different HNSCC-derived CAFs. Relative levels of analyzed genes (*ACTA2, FAP, S100A4, COL1A2, PDGFR α, PDGFRβ, PDPN and POSTN*) were calculated using the 2 − ΔΔCt method; β-actin was used as internal control, and data are presented as fold change relative to NOFs grown as 2D monoculture; *n* = 3 experiments, *p < 0.05 according to student’s t-test

Next, we investigated whether the basal mRNA expression of CAF-associated markers (*ACTA2/α-SMA, COL1A2, FAP, PDGFRα, PDGFRβ, PDPN, POSTN* and *S100A4*) differed between CAFs from different tumors. CAFs originating from tongue (0902, 0843, 0861 and 1002) or larynx (0923, 0820, 0836 and 1001) cancers were cultured in 2D, and the abovementioned markers were analyzed with RT‒qPCR. Our analysis did not reveal distinct differences between tongue- and larynx cancer-derived CAFs regarding the analyzed CAF-associated markers. Overall, four of the analyzed HNSCC-derived CAFs expressed significantly lower levels of *ACTA2/α-SMA* than NOFs. Higher mRNA expression of *FAP, PDGFRβ* and *PDPN* was observed in the majority of the analyzed CAFs irrespective of their origin. The expression of *COL1A2* was generally lower in CAFs originating from the tongue than in CAFs originating from the larynx. Additionally, a significantly increased basal mRNA expression level of *POSTN* was observed only in two of the analyzed CAFs, namely, 1002CAFs and 0820CAFs (Fig. 3).

### Expression of CAF-associated markers in CAFs cocultured with HNSCC cells

To further investigate the consequences of tumor cell-CAF interactions, we cocultured HNSCC cells (LK0412, LK0902 and LK0923) with NOFs and site-matched CAFs originating from different tumors in 2D (0836, 0861, 0902, 0923, 1001 and 1002). After seven days, CAFs/NOFs were separated from tumor cells using MACS, and the mRNA expression of the above-mentioned panel of CAF-associated markers was analyzed and data are presented as the fold change relative to each CAFs or NOF2 grown as monoculture.

**Fig. 4:**
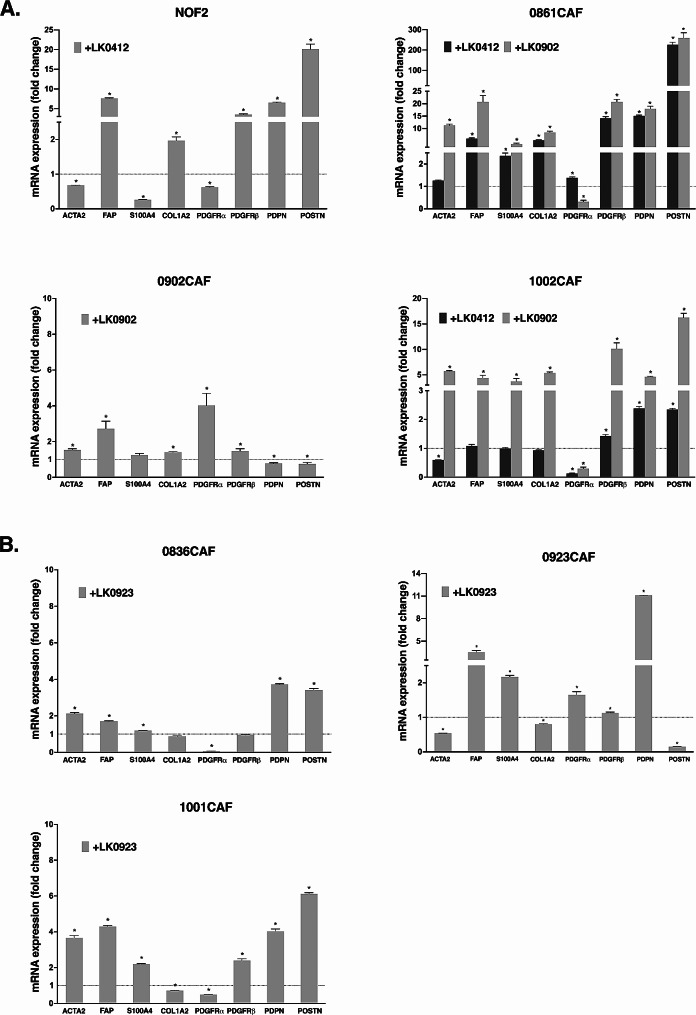
mRNA expression of CAF-specific markers in CAFs co-cultured with HNSCC cells. Three tongue cancer-derived CAFs (0861CAFs, 0902CAFs, and 1002CAFs) and NOF2 cells were cultured with tongue cancer cell lines (LK0412) in 2D **(A)**, whereas three larynx cancer-derived CAFs (0836CAFs, 0923CAFs and 1001CAFs) were cultured with a larynx cancer cell line (LK0923) in 2D (**B**) for seven days and thereafter separated. Relative levels of analyzed genes (*ACTA2, FAP, S100A4, COL1A2, PDGFR α, PDGFRβ, PDPN and POSTN*) in different culture conditions were calculated using the 2 − ΔΔCt method and the data are presented as the fold change relative to CAFs/NOF2 grown as a 2D monoculture; β-actin was used as an internal control; *n* = 3 experiments, *p < 0.05 according to a student’s t-test

The expression of *PDGFRα* was downregulated in almost all unmatched CAFs after coculture with HNSCC cells (Fig. 4). Interestingly, the coculture of LK0902 with their matched CAFs (0902CAF) resulted in only small changes in the expression of CAF-associated markers when compared to 0902CAF grown as a monoculture (Fig. 4A). Moreover, the expression of CAF-associated markers in 0861CAFs and 1002CAFs varied depending on coculture with LK0412 or LK0902, where changes were more prominent. The coculture of NOFs with LK0412 led to increased expression of five out of eight analyzed CAF-associated markers in NOFs (Fig. 4A).

Regarding the cocultures of 0836CAF and 1001CAF with LK0923, the expression of CAF-associated markers was relatively similar in these two CAFs after seven days of coculturing. On the other hand, in tumor matched 0923CAF after coculture with LK0923, the most evident changes were a significant decrease in both *ACTA2* and *POSTN* and a strong increase of *PDPN* mRNA expression (Fig. 4B). Interestingly, the expression of *PDPN* and *POSTN* was significantly upregulated in all six unmatched CAFs and NOF2 cells after coculture with HNSCC cells.

### Expression of CAF-induced genes in HNSCC cells cocultured with CAFs

Our recently published data show that tumor-matched CAFs increased the mRNA expression of *MMP1*, *MMP9* and *FMOD* in tumor cells when cocultured compared to tumor cells cultured alone in spheroids [7]. Thus, we also analyzed the mRNA expression of *MMP1*, *MMP9* and *FMOD* in MACS-separated tumor cells that has been cocultured with tumor-matched and unmatched CAFs or NOFs for seven days.

**Fig. 5:**
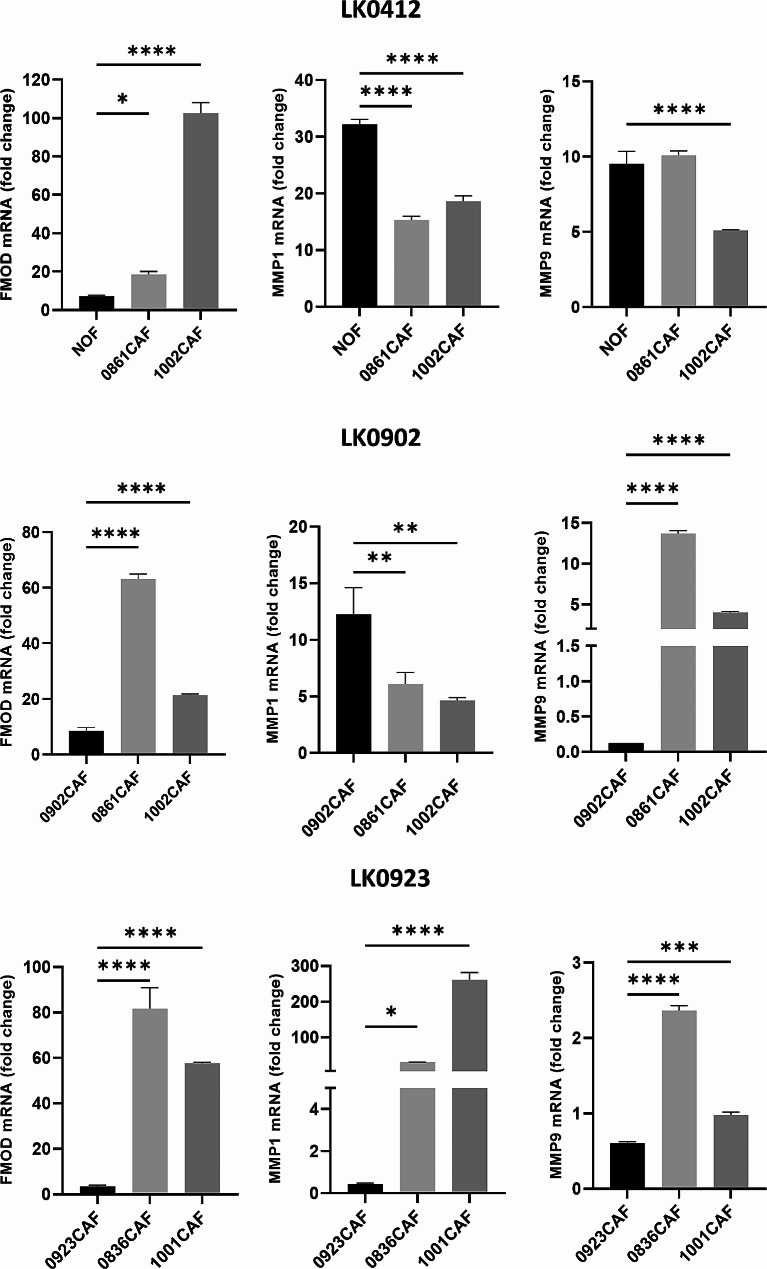
mRNA expression of CAF-induced genes in HNSCC cells after co-culture with CAFs. Relative levels of *MMP1*, *MMP9* and *FMOD* in two tongue cancer cell lines (LK0412 and LK0902) and a larynx cancer cell line (LK0923) co-cultured in 2D with matched and unmatched CAFs for seven days and thereafter separated were calculated using the 2 − ΔΔCt method. Data are presented as the fold change relative to HNSCC cells grown as a 2D monoculture; β-actin was used as an internal control; *n* = 3 experiments. *p < 0.05 according to student’s t-test

**Fig. 6:**
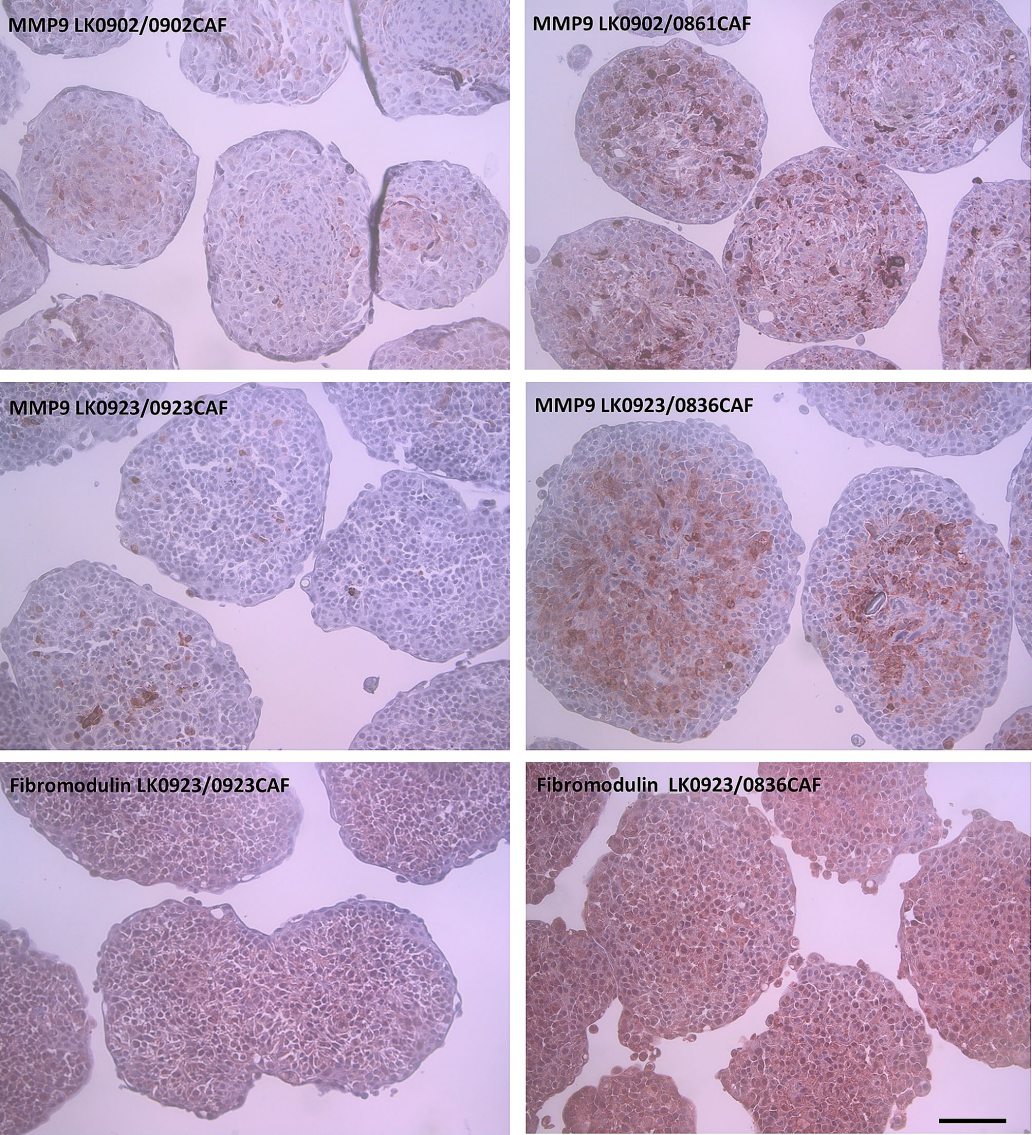
Immunohistochemical validation of *MMP9* and *FMOD* gene expression. Expression of fibromodulin and MMP9 in 7-day-old tumor cells/CAF spheroids by immunohistochemical staining. Scale bar = 100 μm

Our results show that CAFs from different tumors differentially affect the expression of these three genes compared to tumor-matched CAFs or NOFs (Fig. 5). For example, significantly higher mRNA expression of *FMOD* was found in LK0412 tumor cells cocultured with 0861CAF and 1002CAF than in those cocultured with NOFs. Furthermore, these two CAFs also significantly upregulated *FMOD* and *MMP9* in LK0902 tumor cells compared to cocultures with their tumor-matched CAFs. In LK0923 tumor cells, *FMOD*, *MMP1* and *MMP9* were significantly upregulated after coculture with both 0836CAF and 1001CAF compared to tumor-matched CAFs (Fig. 5). To confirm these results, LK0902 and LK0923 spheroids were embedded in paraffin, sectioned, and stained for MMP9 and fibromodulin using IHC. Increased MMP9 expression was found in LK0902/0861CAF spheroids and LK0923/0836CAF spheroids compared to spheroids with tumor-matched CAFs. Furthermore, in LK0923 spheroids, increased expression of fibromodulin was found in LK0923/0836CAF spheroids compared to LK0923/tumor-matched CAFs (Fig. 6).

## Discussion

It is well documented that CAFs are implicated in tumorigenesis and therapy response; however, the mechanism is not fully established [22]. In previous studies, we demonstrated that tumor-matched CAFs cocultured with tumor cells in 3D have an impact on the proliferation and treatment response compared to tumor cells cultured alone in spheroids [18]. Furthermore, CAF-derived signals regulate the expression of multiple genes on tumor cells after coculture [7].

In this study, we investigated whether the impact of tumor-matched CAFs on tumor cells in three HNSCC cell lines is similar to the effect of CAFs from other tumors but originating from the same site or NOFs. Several studies have shown results where tumor cells have been cultured with unmatched CAFs or NOFs [23–25], but to our knowledge, no results have been published where HNSCC cell lines and tumor-matched CAFs have been used. We grew the two cell types in cocultures, allowing cell‒cell contact, and each cell line was cultured with CAFs originating from different tumors but possessing same anatomical localization, NOFs or tumor-matched CAFs.

We first aimed to study how different CAFs affect proliferation in spheroids (Ki-67-positive cells/spheroid area) and found that after coculture of two tongue cancer cell lines with site-matched CAFs, two CAFs affected proliferation, one of which, 0861CAF, increased proliferation in LK0412 spheroids but decreased proliferation in LK0902 spheroids. Furthermore, the expression of CAF-associated markers after co-culture with HNSCC cells was most pronounced in 0861CAF among the analyzed panel of CAFs.

In earlier studies, LK0902 spheroids demonstrated a low number of proliferating cells and furthermore, LK0902 cells were shown to be cisplatin resistant in 2D cultures [18, 21]. In this study, all co-cultures of LK0902 with CAFs showed resistance to cisplatin. In cisplatin-treated spheroids composed of LK0902 and 0861CAF, enhanced cell viability was determined, which was correlated with a low proliferation rate in this co-culture. It is well known that tumors with low proliferation are less responsive to DNA-damaging agents. In a breast cancer study, high Ki67 expression was the only significant factor for predicting a better clinical response to neoadjuvant chemotherapy [26]. Furthermore, in a study of patients with oral squamous cell carcinoma (OSCC) treated with radiotherapy, it was shown that a high number of Ki67-positive cells in the tumor correlated with improved survival [27]. The highest number of proliferating cells was found in LK0923/CAF spheroids compared to LK0902 and LK0412 spheroids, which could be the reason why this cell line is more unaffected by different CAFs or NOFs when looking at proliferation and cisplatin treatment response.

CAFs display great heterogeneity and can have either tumor-promoting or tumor-inhibiting properties. CAFs have also been classified into different phenotypes (basal, mesenchymal, classical or atypical phenotype) due to different mRNA clusters in the tumor from where they were established [23]. To characterize CAFs, the expression of selected markers, such as α-SMA, vimentin, S100A4, CD90, CD73 and CD105, has been used [28], and up to 14 subpopulations of immunostimulatory CAFs have been identified in HNSCC [29]. Other studies have found two distinct subtypes of CAFs with different molecular signatures and tumor-promoting abilities in HNSCC [10, 11]. To examine whether the investigated CAFs have different molecular signatures and how co-culture of tumor cells with CAFs affects their phenotype, the mRNA expression levels of *ACTA2, COL1A2, FAP, PDGFRα, PDGFRβ, PDPN, POSTN* and *S100A4* were analyzed. We did not observe obvious differences between the analyzed panel of tongue and larynx CAFs. In our panel, most CAFs changed their molecular signature after coculture with different tumor cells. Furthermore, when matched CAFs and unmatched CAFs were cocultured with LK0902, only small changes were found in 0902CAF compared to 0961CAF and 1002CAF, where most markers were upregulated. Interestingly, when 1002CAFs were cocultured with LK0412 or LK0902 tumor cells, the effect on the expression of CAF-associated markers was much more pronounced after coculture with LK0902 cells. These results indicate that the interaction between CAFs and tumor cells is likely both tumor cell- and CAF specific.

On the other hand, when NOFs were cocultured with LK0412, five of eight CAF-associated markers were upregulated. NOFs have been used in several studies instead of CAFs to study the impact of tumor stroma on tumor cells. In our study, NOFs have been used in coculture with LK0412, LK0902 and LK0923 to assess their impact on tumor spheroid proliferation when compared with CAFs. Additionally, we have used NOFs in coculture with LK0412 to assess response to cisplatin treatment and to evaluate the expression of tumor stroma-associated genes. Li et al. have shown that in 2D cocultures, CAFs but not normal fibroblasts promote oral tumor cell proliferation [30]. In our 3D model, higher proliferation was observed in cocultures consisting of LK0902/NOF than in cocultures consisting of LK0902 tumor-matched and unmatched CAFs. It is likely that direct contact of NOFs with tumor cells has an impact on the activation status of NOFs and their CAF-like phenotype.

Next, we investigated the mRNA expression of *MMP1*, *MMP9* and *FMOD* in tumor cells after they were cocultured with different CAFs in 2D. Interestingly, we found that both *MMP9* and *FMOD* were upregulated in LK0902 and LK0923 cells when cultured with unmatched CAFs compared to when cultured with tumor-matched CAFs. We have seen in a previous study that tumor-matched CAFs upregulate these genes in tumor cells when cocultured in 3D compared to tumor cells grown alone in 3D. Moreover, we show that high mRNA expression of *MMP9* and *FMOD* has a positive effect on the overall survival of HNSCC patients treated with radiotherapy [7].

A potential limitation of our study is the use of 2D coculture, which is motivated by the direct comparison of 2D mono- and coculture. Nevertheless, we could confirm the mRNA results from the 2D model at the protein level in 3D with IHC. The heterogeneity of CAFs has likely impact on the interaction with tumor cells within the tumor microenvironment and the resulting outcome such as tumor progression and treatment response. However, we have not observed a distinct CAF subgroup regarding the analyzed tumor-matched and unmatched CAFs. Moreover, the mutual interaction between CAFs and tumor cells and its consequence is more pronounced in tumor-unmatched CAFs in terms of cell proliferation and mRNA expression of CAF-specific and CAF-induced genes.

We provide evidence that the crosstalk between the tumor cells and CAFs is essential for influencing tumor progression and therapy response when cocultured in tumor spheroids. Our results highlight the need for further research regarding the mechanism behind the intercellular communication in the tumor microenvironment in the search for tumor microenvironment-derived and clinically relevant biomarkers.

## Conclusions

Taken together, our results indicate that tumor-matched CAFs are unique for each tumor and affect the proliferation and the gene/protein expression of tumor cells in a distinct manner. Furthermore, we show that the interaction between tumor unmatched CAFs and HNSCC cells in the tumor spheroids is associated with significant changes in the mRNA expression of CAF-specific markers and a significant increase in FMOD and MMP9 expression in the 3D coculture model.

## Data Availability

No datasets were generated or analysed during the current study.

## Abbreviations

HNSCC: Head and Neck Squamous Cell Carcinoma
EGFR: Epidermal Growth Factor Receptor
CAFs: Cancer-Associated Fibroblasts
MACS: Magnetic Activated Cell Sorting
PBS: Phosphate Buffer Saline
OD: Optical Density
TBS: Tris-Buffered Saline
